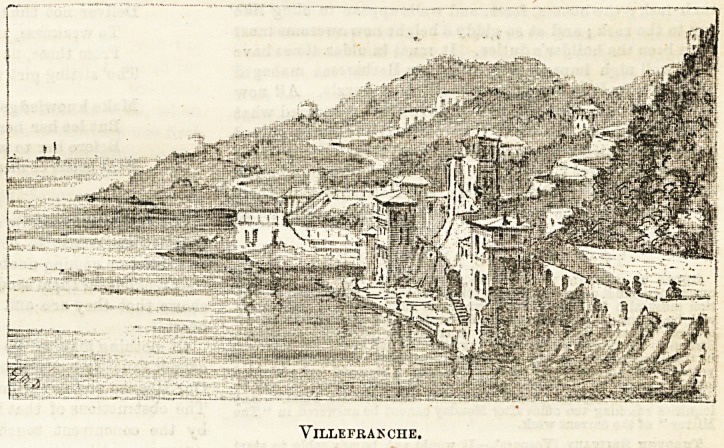# "The Hospital" Nursing Mirror

**Published:** 1899-01-28

**Authors:** 


### "The Hospital" Nursing Mirror.

Being the Nursing Section of "The Hospital."

[Contributions for this Section of "The Hospital" should be addressed to the Editor, The Hospital, 28 & 29, Southampton Street, Strand, London, W.C., and should have the word "Nursing" plainly written in left-hand top corner of the envelope.]

### News from the Nursing World.

### THE ETIQUETTE OF SPAIN.

The Spaniards have a reputation for pride, which, if this reported little tale be true, is fully maintained by the courtiers of to-day. The handsome young wet-nurse of the baby king was discovered by the Duenna committing the enormity of kissing her charge, and further added to her fault by excusing herself "because she loved him." The ladies in attendance were horrified, and upbraided the poor thing so vehemently that she burst into a flood of tears. Fortunately for both nurse and baby the Queen Regent took a more humane view of the matter, and instead of severely reprimanding the culprit, as her ladies expected, said kindly, "Of course she wants to kiss her nursling," and further declared that the matter was one in which "etiquette must be forgotten!" Poor ladies! It reads like a fairy tale, yet, nevertheless, it calls to mind the etiquette of the French Court which kept the poor Queen shivering whilst the ladies of the toilette handed her garments from one to another according to their respective ranks.

### SIR SQUIRE BANCROFT'S ART.

The delightful art of story-telling is not dead. Any-one who is doubtful about the matter cannot have heard Sir Squire Bancroft "read" Dickens' "Christmas Carol." It is a simple tale of humble folk, with stagey ghosts and burlesque humour. Yet how human is the interest that enfolds it, how divine the lesson it enshrines! And the story gains greatly in the telling. Without apparent effort the trained voice flows on in sympathetic modulation until the magic of the story lays its spell upon his audience, and for the space of two hours the present is forgotten, and Scrooge and his experiences are realities. Sir Squire Bancroft has added largely to the funds of various charities by his popular "readings," and on Thursday afternoon last the City Hospital for Diseases of the Chest benefited directly and indirectly (by donations) to the sum of £300.

### MIDWIVES' INSTITUTE AND TRAINED NURSES' CLUB, 12, BUCKINGHAM STREET.

The annual general meeting of members of the Midwives' Institute and Trained Nurses' Club took place on January 20th, and was, as usual, the occasion for a very pleasant social gathering. In spite of wretched weather there was a good attendance of members, and a most satisfactory account was put before them. The amount of solid work accomplished by this nursing centre increases year by year, and its council and committee are much to be congratulated on it. The training for midwives has proved entirely successful, and has been much appreciated by those who have profited by the excellent lectures and classes, four courses of which, each consisting of 26 lectures, have been held at the club during 1898. The meetings of "Nurses in Council" have resulted in very interesting discussions on subjects of special importance to nurses. The Society of Trained Masseuses (also associated with the club) reported a good year's work. The number of members now holding this society's certificate is 151, and the number of applications for masseuses continually increases. Financially the past year has been a good one. The announcement that Miss Wilson had consented to stand for re-election as president was received with much satisfaction, and she was unanimously elected to the office for the coming year. After the business was concluded the members were invited to tea by the treasurer, Miss R. Paget, and a very pleasant evening was spent.

### THE SALARIES ACT IN IRELAND.

Under the Local Government (Ireland) Act the Guardians of Ireland are entitled to claim the return of half the sum paid in salaries to the trained nurses in their workhouses. It is undoubtedly a mighty weapon in the hands of the Local Government Board against the employment of untrained women, and will open the eyes of many to the advantages of competent service in the nursing department. The South Dublin Union is one of the first to receive official notification of the terms on which the Board may apply for the return of the money. The Guardians, in commenting upon it, showed that there is already a cavilling spirit abroad at the definition of the term "trained" nurse being interpreted by the Local Government Board "certificated" nurse. Of course, the latter term does not include the members of various Sisterhoods who do so much of the Poor Law nursing in Ireland. It is the fate of concessions to be met with discontent, and, although it is thoroughly illogical in the present instance, yet many a battle will be fought and won before the righteousness of the determination that paupers shall have nurses whose skill is certified is apparent to those interested in the prevailing system. It must be clearly understood that the local relief to the rates effected by this Act was granted in response to the argument, "You compel us to employ certificated nurses, and we cannot pay them." It is absurd, therefore, that, when this plea is met by legislation which practically says, "We will pay the difference," an accusation of injustice should be raised against the central Board because it limits its bounty to those who employ nurses who hold certificates.

### FOR THE NURSES OF INVERNESS INFIRMARY.

The designs of the new nurses' home to be erected by the managers of the Northern Infirmary, Inverness, have been prepared by Messrs. Ross and Macbeth. They represent a picturesque cottage residence, containing eight bedrooms on the upper, and five bedrooms, kitchen, and sitting-room on the ground floor. The site is behind the porter's lodge, and the home will face south, overlooking the gardens. The cost is estimated at £1,472.

### LICHFIELD NURSES.

The annual meeting of the General Committee of the Lichfield Nursing Home took place recently at the Guildhall. The income for the year was £200, and the expenditure £192. Measured by this standard the donations and legacies to the association have been munificent, and the only thing to be said about the proposal to set to work at once to build the nurses' home, so long in contemplation, is that it might have been done long ago. In the first place, the Jubilee Fund placed £612 to the credit of the association; Mr. Seckham offered £500 and a site; and Colonel Wilkinson £100; whilst not so long ago the association received £20 a-year from the residuary estate of Mrs. Mary Slater until the death of Mr. Brown, when a larger portion will fall to its share. It was suggested that the home shall be erected on the site offered by Mr. Seckham; but prior to taking definite steps all the proposed sites will be reconsidered, let us hope with more profitable results than heretofore.

### CERTIFICATED POOR LAW NURSES.

The Norwich Guardians are tackling the nursing difficulty with a spirit and a determination that promise success in some shape or another. Our readers may remember how disappointed they were when the authorities of the Norfolk and Norwich Hospital, whilst allowing the workhouse probationers to attend the hospital lectures, rightly refused to grant them certificates. They have now hit upon a plan—upon which they are consulting other guardians—which, if favourably regarded by the Local Government Board, would influence in a far-reaching manner nursing affairs in the smaller unions. It is suggested that the Local Government Board should be approached with a petition praying that that Board shall establish periodical examinations at convenient centres where probationers may sit for examination in poor law work, and that the Board shall grant certificates to successful candidates which shall qualify them for the post of superintendent nurse. It is also desired that the article (Article 3, Section 3), which insists upon the medical officer being "resident," shall be dispensed with. It is also proposed that this grade of nurse shall be known as "Certificated Poor-Law Nurse." The position of the Local Government Board in dealing with the whole question of poor-law nursing is diametrically opposed to this proposition. The Board, so far, has deliberately refused to recognise the training given in the smaller institutions, and with good reason. The aim of the order of 1897 was to establish a system of fully-trained nurses in the workhouses, and not to recognise inferiorly-trained women as good enough for the work. It is manifestly impossible that a small workhouse can be so fully equipped either in staff, appliances, or cases as to afford equal opportunities with the larger infirmaries and hospitals for the training of nurses. But the great reason against the establishment of such a grade of nurses as is proposed is that their certificates would be obtained by examination rather than by training. Therefore, if the Norfolk proposal were carried, the result would be that an inferior grade of nurse would be evolved which the Local Government Board does not want, and which the workhouses are as well without.

### UNTRUE ACCUSATIONS.

It is only fair to the management of the Woodbridge Isolation Hospital to make known the result of the recent inquiry into the accusations of Nurse Hartland. The charges were that the matron had made her a subject of gossip amongst her fellow-workers; that she had had to "sleep in the diphtheria tent and live in a scarlet kitchen"; and that there was not enough bread. As regards the first, there were plenty of witnesses to declare that no interest was taken in the alleged gossip, and that when Nurse Hartland herself introduced the topic of her testimonials no one paid any attention to her. As regards the second, the "scarlet kitchen" referred to was the nurses' observation ward in the scarlet fever block, the nurse's proper place when there were (and there were none at the time) cases to be nursed; and that the "diphtheria tent" was a small room opening out of the lobby of the building for this disease between the two wards; it was surrounded on three sides by open passages, and it adjoined on the fourth the outer ward. There were no cases in during Nurse Hartland's occupation, though a suspected case was under observation during the last week in December. The bread supply was abundant, and the evidence appears to show that the nurses and servants are comfortably catered for and housed. The fact that a new administrative block was in course of completion, which when finished would have removed any inconveniences, should have deterred criticism at the moment, and the board's prompt investigation of the complaint is satisfactory in every way.

### BULWELL NURSING ASSOCIATION.

After mature consideration and careful preparation, a public meeting was held at Bulwell on the 16th inst. to take the first steps to inaugurate a nursing association. The Rector presided, and, ascribing the credit of the movement to Mr. Hill and Mr. W. H. Carey, he announced that already in addition to promised donations they had a subscription list of £150 a year, and he thought they could easily support two nurses, and save up for a home in due time. Mr. Ellis, in speaking to the resolution, said that the majority of inhabitants were colliers, and that the friendly and benefit societies had been approached, with the result that half their subscriptions came from this source. A representative would be placed on the General Committee for each £5 contributed. A Ladies' Committee had also been formed. Sir Charles Seely having been appointed president, and the committee elected, the proceedings terminated.

### SHORT ITEMS.

The nurses' home that is being built at the New Hospital for Women, Euston Road, will be opened on February 1st by the Bishop of London.—Half the proceeds of the performance of "The Messiah," which was given last week in the Town Hall, Loughborough, by the local musical society, will be given to the Queen's Nursing Association. The other half will be sent to the Charnwood Forest Children's Convalescent Home.—A large increase in the nursing staff is necessary to bring the requirements of the West Bromwich Workhouse up to an efficient standard. The recommendations of Dr. Fuller involve an expenditure of £20,000, and include the appointment of an assistant medical officer and other officers.—A ball was held in the Town Hall, Blackburn, on behalf of the District Nursing Association, on the 12th inst. Over 300 guests were present.

### Hints on the Home Nursing of Sick Children.

By J. D. E. MORTIMER, M.B., F.R.C.S., formerly Surgical Registrar, &c., at the Hospital for Sick Children, Great Ormond Street.

(*Continued from page 169.*)

FEEDING.

As mentioned in my introduction, I have not attempted to deal at length with the wide subject of the feeding of infants and young children. The nurse, if in any doubt, should ask the doctor for precise directions. There are, however, certain points to which attention may be drawn, as they become of increased importance during illness and convalescence. In case of infants there is necessity for the utmost cleanliness, accurate preparation and measurement, and proper temperature. I need hardly say that no feeding-bottle with a tube is admissible. The rubber teat must be thoroughly cleaned inside as well as outside, and if new should be well soaked before using. Milk must be kept protected from dust in a cool place free from smells. "A room warm enough for a bed-room is too warm for a larder." Remember that binders, &c., which seem right before a meal may be uncomfortably tight afterwards. "Jigging" babies on the knee is a frequent cause of sickness. A baby should never be allowed to lie flat on the back during or soon after a meal.

In administering food it may be necessary to restrain the movements of a "troublesome" baby by wrapping it from head to foot in a blanket, and the nurse should see that there is nothing distracting its attention nor causing discomfort. Sucking may be interfered with by some obstruction in the nose, which obliges the baby to breathe through the mouth, or by soreness in the mouth; there will in such cases be repeated attempts, the infant desisting with a disappointed cry. If the throat is sore, the nurse may notice that the food is taken with a gulp, followed by a cry of pain. An abscess or other swelling at the back of the pharynx is another cause of difficulty.

After infancy, milk in some form should still be the staple food for some years. Young children should not have fried meat, salted or otherwise preserved meat or fish, highly-seasoned dishes, new cakes, pastry, pickles, nuts, or anything similar. Vegetables must be tender and well cooked. Soft fruits are quite allowable, but not those containing hard angular seeds such as raspberries, currants, blackberries; the pulp only of stone fruit should be given, and apples are best cooked. No sweets, cakes, &c., should be allowed between meals—they destroy the appetite, and it is impossible for the digestive organs to do their complicated work if they are continually worried by having a fresh task set them before the last one is disposed of.

In ordinary health and in convalescence children are often given too much meat, and most people have a very exaggerated idea of the virtues of "strong beef tea." This may be useful to a certain extent, but it cannot replace other kinds of food, and to many children, especially to the thin and nervous, is positively harmful. Some of the meat essences so much advertised are of little or no value (except in a commercial sense), and merely give the system the trouble of getting rid of them.

Whilst it is very bad policy to encourage "fads" or to yield to mere perversity, it must be allowed that young children have sometimes, towards quite ordinary articles of diet, a perfectly genuine and uncontrollable dislike, which is only made all the stronger, even to actual vomiting, by direct opposition, but wears out in time if left alone. Want of proper training also creates difficulties in illness, and at such time is better met by diplomacy than by sudden efforts at reform. For instance, some children "cannot take" milk, but will drink "milk and water," or it may be given as custard, junket, chocolate, or flavoured and stiffened with isinglass. Cream or fat bacon may be given instead of cod-liver oil and so on.

Sometimes (as at the outset of fever) it may be wiser to give very little nourishment, especially if there is distaste for it, as it is likely to be rejected, or if retained, merely to add to the little patient's troubles. It also seems to me worse than useless to force down nourishment in the later stages of hopeless disease. If there is vomiting nothing should be given for an hour or so, and then only cold water, a teaspoonful at intervals; if this is retained it may be replaced by whey or barley water, with milk gradually added. The milk may be peptonized by Benger's or Fairchild's method, or some preparation such as Horlick's or Savory and Moore's prepared milk given. Brandy or other stimulants, if ordered, should be well diluted and given in small quantity at frequent intervals. Their apparent effect on the strength and regularity of the pulse must be observed, and caution is needed if there is drowsiness, vomiting, or delirium.

In most cases nourishment, including milk, which should be regarded as a food and not as a drink, is not required oftener than every three hours. It is a mistake to tease the patient and interfere with appetite and digestion by continually urging trial of a little of this or that. But in special cases the doctor should be asked to write down exactly the amount and kind of food, and how often it should be taken, and also whether the child is to be awakened, and the nurse must do her best to follow the directions. If the mouth is hot and clammy it should be first cleansed. If the amount of drink is limited no more should be offered than the child is to be allowed to take, and it should be put into a small cup or tumbler. At night, and in cases of exhaustion, a feeding-bottle or feeding-cup should be used to avoid disturbance.

NASAL FEEDING

is done with a funnel and soft rubber tube (lubricated with glycerine and water, not oil, which spoils it). The tip of the nose is slightly pushed up, so that the nostrils look rather forward, and the tube gently introduced along the floor of the nose, not upwards. If there seems to be any obstruction on one side the other should be tried. When the first three inches have passed it may be pushed on more quickly. If there is insensitiveness of the larynx there may be little or no coughing if it goes into the windpipe, but there is a sudden check, and the child becomes more or less cyanosed, whilst on applying the cheek to the funnel air may perhaps be felt to issue from it at intervals corresponding to the breathing. It must, of course, be withdrawn, and another attempt made. When the tube is in the right track it may be felt to be in the grasp of the gullet, but there is no obstruction, and its whole length passes easily; the child, in fact, swallows it as it is paid out, not as when it is coiling up in the mouth or has become doubled. On entering the stomach some gas may escape, but irregularly. It must be understood that this operation should never be attempted without proper assistance to hold the child firmly (see later on), otherwise the head may be jerked away and, the tube being half out, the food may enter the windpipe and cause fatal choking. Nor should it ever be undertaken by anyone who has not already practised it under the supervision of some qualified person. In any case, only about a teaspoonful of fluid should be first poured into the funnel. If this does not go down, owing to air in the tube, the funnel should be tilted or slightly shaken, or the tube squeezed between the finger and thumb in an upward direction.

(*To be continued.*)

### Nursing in Enteric Fever.

By WARREN G. WESTCOTT, L.R.C.P.Lond., M.R C.S.Eng., Resident Medical Officer, Chichester Infirmary.

(*Continued from page* 137.)

THE all-important subject of diet in enteric fever calls for careful consideration. There are but few other conditions in which it is of so vast importance. Many cases have severe complications, suffer relapses, or end fatally, in consequence of indiscretion in diet, and when one considers what the state of the alimentary tract is, it must be apparent how easy a matter is it to aggravate it by improper aliment. All solid food must be strenuously interdicted from the very commencement of the illness up to and for at least ten days beyond the appearance of morning and evening temperature at the normal line. Fresh cow's milk should be the staple article of nourishment throughout the whole course in all cases, whether mild or severe; it may be given diluted or undiluted, boiled or unboiled, in both cases the former being frequently better retained. Meat extracts, juices, and essences, together with similar preparations, may also be given unless contra-indicated. All these, however, take but a secondary place in regard to value as nutritive agents, and act beneficially chiefly by virtue of their stimulating properties, doing much good in selected cases. In giving any particular food one must not forget that the stomach shares with all the organs and tissues of the body the loss of tone so marked in this particular disease, and that assimilation is consequently less perfectly carried out. It is therefore most important to avoid overloading it. At the same time the system needs more than the usual amount of nutriment to counterbalance the extensive tissue waste and to support the vital functions, consequently one may say that as much as the patient can take and at the same time assimilate does good—more than this, harm. In order to help the stomach to carry on its important duties food should be given in small amounts, at frequent intervals, the exact amount with which to commence being determined by the age and general condition of the patient. For a child of, say, 10 years the following would be a reasonable quantity: Milk, ℥iiss. every two hours; beef tea, ℥iiss. every six hours, *i.e.*, about Oij. of milk and Oss. of beef tea in twenty-four hours. For an adult a fair amount would be: Milk, ℥v. every two hours; beef tea, ℥v. every six hours, *i.e.*, Oiij. of milk and Oj. of beef tea in twenty-four hours. As an indication that too much work is being thrown upon the digestive organs there will probably be sickness and perhaps diarrhœa, with other abdominal discomfort. On examining the vomited matter one will often find curds (precipitated casein), and if there happens to be diarrhœa a similar condition in the stools. The indication is to diminish the amount of milk ingested, and at the same time to dilute it, if this has not already been done. As diluents one may employ lime, potass, or soda water, which prevent the formation of curds by neutralising the abnormal gastric secretion. Barley water may be used instead; this acts partly as the above, but principally, owing to its bland properties, soothing the gastric and intestinal mucous membrane. The proportion of milk to the diluting agent should be as 5 to 2. By one or other of these means the result sought for is obtained in most cases; milk given to adults is often more palatable and taken better if flavoured with tea or coffee.

Such preparations as beef-tea, chicken or veal jelly, should as in the administration of milk, be given cold. Jellies may be given in that form or liquified, and in all cases only small amounts at a time. For thirst, mucilaginous drinks or toast water, or acidulated drinks, as lemon water, are grateful, and may be given freely if causing no discomfort. Stimulants are needed at some time of the illness in most cases, often from the very beginning in debilitated subjects, whilst in children there may frequently be no necessity for them. The indications for their use are increasing heart weakness, as shown by a feeble and rapid pulse, and marked nervous prostration, in which low muttering, delirium, and the general typhoid state are seen. Brandy is usually chosen; this, for children, should be freely diluted and sweetened. Adults often do better with good red wine as Burgundy or old port, whilst a sparkling wine as champagne may succeed best when the leading feature is extreme prostration, especially in elderly patients. Whatever form be decided upon, it should be given regularly, in small amounts, and at frequent intervals, either in the milk or beef tea; if separately, well diluted. For a child, brandy, ʒi.-ʒii, or more according to necessity every two hours; to an adult ʒii.-℥i. or more, the exact amount in any case being determined by the age of the patient and the cause for which prescribed.

Leaving now the subject of diet, there are various circumstances which may arise and give cause for anxiety if continued. A very common one is a temperature which keeps up too high, say 103·5 deg. or over, morning remissions being only slightly marked; here, in addition to the usual sponging, it should again and again be employed for 15 minutes, and the temperature afterwards taken and registered on the chart by an interrupted line; this proceeding will usually bring the temperature down temporarily 2 or 3 deg.; if, however, but little effect be produced, cold or ice-cold water may be used, and in those cases of hyperpyrexia, where it is imperative that it be brought down if possible, the cold pack or immersion for 15 minutes in a bath of water at 90 deg. rapidly cooled to 60 deg. with lumps of ice. This, however, is unadvisable as a method of treatment in those cases of great cardiac weakness with high temperature, but gives excellent results in hyperpyrexia with extreme nervous prostration. Prolonged high temperature is detrimental as by it tissue waste is much increased. This is already very great in enteric fever, and so further cause of such should be combated with. Sickness more or less marked is often present quite early. It is, as already mentioned, usually due to the ingestion of too much food, or of an improper kind—the remedy has also been alluded to; frequently it will be found that milk, given in whatever form, as diluted, boiled, or peptonised, is rejected when cold, whilst if given warm it is retained. In some cases milk has to be discontinued altogether for a few days, owing to constant rejection, and in these cases it is necessary to give the needful amount of proteid material in some other way, as, for instance, egg albumen in water, flavoured with lemon juice—the white of one egg to every two ounces of water. By continuing this for a short time milk is usually afterwards retained without difficulty; if still sickness be present, rectal feeding must be resorted to by means of nutrient suppositories or enemata; the latter should be small in amount, not more than ℥v. at a time, and should be given every four hours; a simple soap and water enema must be administered once in the twenty-four hours, with a view of clearing out the lower bowel. The digestion and assimilation of nutrient enemata may be assisted by peptonising the milk. Sickness as a symptom of peritonitis will be mentioned later.

(*To be concluded.*)

### KINDNESS TO NURSES.

MR. HENDERSON, of the Grand Theatre, Fulham, has very kindly offered to allow the nurses of the infirmary and workhouses to see the pantomime free of charge.

### Training in the Provinces.

(*Continued from page* 170.)

### ROYAL SOUTH HANTS INFIRMARY (114 beds).

### Terms of Training.

Two classes of probationers are received for training at the Royal South Hants Infirmary, Southampton: (*a*) One year probationers, paying an entrance fee of one guinea, and a training fee of 30 guineas, payable half-yearly in advance; and (*b*) three-year probationers, paying an entrance fee only of one guinea, giving their services gratuitously for two years, and receiving a salary of £10 for the third year. The age considered desirable for candidates is between 21 and 35, preference being given to those between 23 and 30. Good education and sound health are essential qualifications.

Candidates enter for a probationary period not exceeding three months, at the expiration of which time, if approved, they sign an agreement binding them to the service of the infirmary for the specified term of training. In their third year probationers are called staff probationers, and have a slight difference in uniform to distinguish them from the first and second year nurses. Staff nurses, who are always promoted from amongst the probationers, and of whom there are only three (one in the out-patient department, one in the male, and one in the female accident and surgical wards), receive a salary of £20 per annum, with uniform and laundry. The appointment is for one year. Sisters' salaries range from £30 to £35, and they, like the rest of the staff, are provided with indoor uniform and laundry.

Probationers are put on night duty for three months at a time. The work and hours of the paying probationers are precisely the same as those of the regular probationers, but they are not required to take night duty, except under special circumstances at the discretion of the matron. Lectures are given to the nurses by three members of the visiting staff, who hold examinations at the close of each set of lectures. The matron also has classes for the probationers.

### Hours On and Off Duty.

Probationers go on duty in the morning at 7 a.m., and leave their wards in the evening at 9. Between 9 and 10 a.m. they are allowed half an hour for luncheon, and dressing, &c.; they have two hours daily for recreation, besides time for dinner and tea. Leave of absence is given one day a month from 2.15 to 9 p.m., and probationers are off duty on alternate Sundays from 3 to 9 p.m., or from 10 a.m. to 3 p.m. They are expected to attend chapel, when they can be spared from the wards, on Thursday afternoons, and on the morning of their Sunday onduty. The daily two hours "off" are either from 10 a.m. to 12 noon, from 3 to 5 p.m., or from 7 to 9 p.m. Sisters are on duty between 8 a.m. and 9 p.m. Besides meal hours they are off duty from 3 to 5 p.m. two afternoons in the week, 3 to 6 one afternoon, and 6 to 10 p.m. on three evenings in the week. On alternate Sundays they have leave from 3 to 10 p.m., and one whole day off duty in the month, with leave from Saturday to Monday once in five weeks.

Annual holiday is for probationers and nurses a fortnight or three weeks; for sisters four weeks, of which one week can be taken in the winter if desired.

Night nurses are on duty between 9 p.m. and 9 a.m., taking their exercise and recreation either from 9.30 to 11.30 a.m. or from 5.30 to 8.30 p.m., according to the time of the year.

### Meals.

The day staff breakfast at 6.30 a.m.; they have lunch between 9 and 10; dinner at 2.15; tea, 5 to 5.30 p.m.; and supper at 9.10 p.m. The ward sisters breakfast in the nurses' dining room at 7.40 a.m., they have dinner, for which three-quarters of an hour is allowed, at 1.30 p.m., and their tea and supper are served in their own sitting-rooms. Night nurses breakfast at 8.30 p.m., their "teas" are taken at 12 30 a.m. and 4 a.m., and they dine when they come off duty at 9.10 a.m.

---

### TAUNTON AND SOMERSET HOSPITAL (100 Beds).

### Terms of Training.

Ladies, and suitable women of every class, are received for training at the Taunton and Somerset Hospital, Taunton, on a four years' engagement. One month's trial is required of candidates, whose age should be between 21 and 30. No payment is expected from ordinary probationers, who begin during their first year with a salary of £5. The second year they are styled "assistant nurses," and are paid £15; nurses in their third year receive a salary of £20; and in their fourth year, as "certificated nurses," £28 per annum. During the first and second years nurses receive continuous training in the wards, during the third and fourth years they are employed, as r quired, either in hospital or in private nursing. Indoor and outdoor uniforms are supplied by the hospital, and a reasonable amount of washing.

A limited number of paying probationers are admitted for periods of three months on payment of thirteen guineas. Board, lodging, and washing are provided for them, but they are expected to supply their own uniform. If their work has given satisfaction, and they wish it, paying probationers may be transferred to the regular staff. If a nurse wishes to break her engagement before the expiration of the four years, she is required to give three months' notice, and to pay a forfeit of £5.

Night duty is taken for three months at a time. There is a permanent night superintendent. Lectures are given to the nurses during their training, and examinations are held yearly.

### Hours On and Off Duty.

Day nurses and probationers go on duty in the morning at 7 o'clock, and their day's work ends at 9 p.m. During these hours two are allowed daily for recreation, besides time for meals, and a quarter of an hour for dressing after the early morning's work, with ten minutes for lunch. Leave is given once a month from 10 a.m. to 10 p.m. (the whole day is given if a nurse has earned it by punctuality, but it is not a rule), and four weeks' annual holiday is granted to all the staff as the lady superintendent may be able to arrange. Sisters are on duty in their wards from 8 a.m. to 9 p.m. They are entitled to two hours off duty daily besides meal times, to a half holiday, from 2.15 to 10 p.m., once a month, and leave to 10 p.m. every fourth Sunday.

Night nurses go on duty from 9 p.m. to 9 a.m.; they take their recreation from 10 a.m. to 1 p.m.

### Meals.

All meals except early lunch and the night nurses' midnight meal are served in the dining-room. Butter is served out to each nurse twice a week. The hours of meals are as follows: Day nurses and probationers breakfast at 6.30 a.m., dinner is at 1.15 or 1.45, tea at 4 or 4.30, and supper at 9 p.m. Sisters breakfast at 7.45 a.m., their dinner is at 1.15 or 1.45, tea 4.30 to 5 p.m., and supper at 9. Night nurses breakfast at 8.30 p.m. and dine at 9.30 a.m.

---

### ROYAL ALBERT EDWARD INFIRMARY, WIGAN (132 Beds).

### Terms of Training.

Three years' training is offered to "women of education and refinement" at the Royal Albert Edward Infirmary.

Candidates should be not less than 22 nor more than 35 years of age; they enter, in the first instance, upon a two months' trial, during which period of probation they are required to provide themselves with the hospital uniform. If accepted as probationers they sign an agreement to serve the hospital for three years, dating from their first entering. No premium is asked from ordinary probationers, whose pay begins at a salary of £12 for the first year, rising to £14 the second and to £16 the third year. Sisters' salaries range from £27 to £31 per annum. All the regular staff are provided with full indoor uniform and with laundry by the hospital.

A complete course of three years' training is an essential qualification for promotion to the post of ward sister, either in the hospital itself or at some other accredited training school.

A certain number of paying probationers are received for periods of not less than three months on payment of £6 5s. for such period. The age considered desirable for paying probationers is the same as that already mentioned, and no difference whatever is made between the ordinary and the paying probationers as regards their work. The paying probationers perform the same duties, and are allowed to attend the lectures and take advantage of the technical teaching given to the regular staff by the doctors and the matron. A certificate is granted at the end of a year.

Night duty is taken for three months in each year by the probationers, supervised by a permanent night sister.

Hours On and Off Duty.

The working day for all the nursing staff is some ten and a half hours, excluding meal times and off duty hours. The day nurses go on duty in the wards at 7.30 a.m., and leave them in the evening at 9 o'clock. Two clear hours are allowed daily for recreation, and half an hour each for dinner and tea. A whole day's leave is given once a month, and, as a rule, on these occasions nurses can leave the hospital the previous evening, so that they may get all the good possible out of their holiday. On alternate Sundays leave is given from 9.30 a.m. to 12.30 p.m., or from 5 to 9 p.m. Annual leave is—for probationers two weeks, for nurses and assistant nurses three weeks, and for sisters one month.

Meals.

All meals, excepting luncheon, are served in the dining hall; no "allowances" are made to the nurses, everything needful being provided for each meal. The day staff breakfast at 7 o'clock; they have their early lunch about 8.45, in the ward kitchens; dinner is at 12.30 and at 1.15, tea at 4.30 and 5, and supper at 9 p.m.

### Chelsea Hospital for Women.

### OPENING THE NEW NURSES' HOME.

On Friday, January 20th, the Duchess of Albany, attended by Sir Robert Collins and the Hon. Mrs. Moreton, opened the new home in Neville Street recently acquired for the probationers of the Chelsea Hospital for Women, Fulham Road, S.W. Her Royal Highness, who was dressed in silver-grey silk, with white waistcoat and revers, looked very well. She was received at the home by Mr. F. Dyer Edwards, J.P. (the vice-chairman), Sir W. J. Colville, Mr. Henry E. Wright (the treasurer), Dr. William Duncan, Miss Heron-Maxwell, and Miss Heather Bigg (the matron). Several members of the ladies' committee and the secretary were presented to her in the superintendent's room, and afterwards the visitors inspected the building. They then proceeded to the hospital, where many friends of the institution were assembled in the board-room to welcome the Duchess, and Miss Gladys Duncan presented her with a bouquet of pink roses. Amongst others were Lady Florence Pelham Clinton, Lady Wilfreda Biddulph, Lady Colville, the Countess of Gosford, the Countess of Iddesleigh, Lady Howard de Walden, &c.

The Vice-Chairman made a short statement of the improvements now in progress, and for which a sum of £4,000 is necessary. The lease and alterations of the new home have cost £1,600, and the furnishing £400; in addition, it has been found necessary to enlarge the operating theatre, to put in a lift, and to finish the installation of the electric light. For these objects £1,000 has already been received, including a donation of £100 each from Dr. and Mrs. Fenton, and £50 from Mr. Henry E. Wright.

The Duchess then visited the wards, which looked very bright and comfortable, and were lavishly adorned with flowers in honour of her visit. She spoke to each patient, giving her at the same time a small breast-knot, which was handed to her for the purpose by Miss Duncan. Her gracious and kindly manner left a very pleasant impression on everyone. One patient, who was to leave the hospital the next day, congratulated herself that she had been able to stay so long; and another, who was about to undergo an operation, was encouraged by having her flowers selected from the Duchess's own bouquet. The matron had every reason to be pleased with the interest exhibited in the domestic mechanism of the establishment, for the Duchess descended into the basement with an animated expression of housewifely sympathy.

After tea in the matron's room the Royal party drove away, and some of the guests went to the home.

This is a house of an ordinary residential type, consisting of three storeys and a basement. On each floor there are two rooms, the front one being divided by a stained and varnished wooden partition into two fairly large bedrooms. In accordance with a decree of the County Council the solid partitions were not allowed to reach the ceiling, because there was only one fireplace for the two rooms. Consequently, the portion remaining between the wood and ceiling has been filled with trellis-work. One little room on the half-landing has been fitted as a bath-room, which is well supplied with hot and cold water.

The dining-room is a large, comfortable room in the basement, and is only used for breakfast, dinner and tea being provided at the hospital. The kitchen is good, and the scullery has been converted into the servants' bedroom. The house is well furnished throughout, the suites consisting of combination wardrobe and a marble-topped washstand of ash. Vanity as regards the toilet is discouraged, a small hanging glass only being provided for the adjustment of the headgear. Altogether the home is very comfortable, the chief drawback, of course, being the distance between it and the hospital, which was unavoidable. It is, as before remarked, for the accommodation of the probationers, who number about a dozen, and who sleep, breakfast, and spend their off-duty hours in it, their meals, work, and instruction being provided in the hospital.

The home is under the superintendence of Miss Dearman, who is untrained as a nurse, but who has had much experience in institutional management. She will also assist the matron in her housewifely duties.

### The Pension Fund Nurses.

### MISS BURNS' WEDDING GIFT.

We have received 6d. from Nurse S. A. Pittman, who writes that she has been away and so has not heard until the present of this subscription.

### A Book and its Story.

### "THE DAY'S WORK."

Gladly as we welcome a new book* by Rudyard Kipling it is always with a certain feeling of disappointment—a feeling that perhaps is ungrateful considering what he has given to us, but a feeling that is shared by those who love his writings best, and still more by those who have watched him since his early days in Anglo-Indian journalism. We recall the promise he gave us in "Plain Tales from the Hills" in his early youth, and we look in vain for the fruition of that promise in his later works. With sorrow it must be confessed that Kipling's best work was done at an age when the subaltern whom he loves so well is learning his duty as orderly officer, and his subsequent writings show, if no falling off, still no advance. The man who could create Mulvaney at the age of twenty, what greater conceptions has he given us in his maturer years? A stanza or two that ring and will remain, and the rest disappointment. Popularity too easily earned has robbed us of the finest writer of the century.

"The Day's Work" is a collection of short tales, 13 in number, the majority of which have appeared before. Each has its peculiar interest, and shows the wonderful power of the writer in depicting scenes, his knowledge of men in all parts of the globe, and his keen sympathies with animals.

To review all the tales separately would take up too much space, and we shall therefore confine ourselves to what we think the best in the collection—though doubtless many of our readers will consider, and with good reason, that one or more of the other tales in the book have equal claims to attention. The scene of "William the Conqueror" is laid in India—partly in Lahore and partly in Madras—during a famine. The story opens at the Punjab Club in Lahore, where one evening in the hot weather three men are found discussing the prospects of an outbreak of famine in the Madras Presidency. The news arrives that the famine has been duly "declared," and Scott, of the Irrigation, and Martyn, of the Police, presently receive their orders to proceed on relief works to the south. "William," Martyn's sister, accompanies her brother. "Miss Martyn had come out to India four years before to keep house for her brother, who as everyone again knew had borrowed the money to pay for her passage, and that she ought, as all the world said, to have married long ago. Instead of this she had refused some half-a-dozen subalterns, a civilian twenty years her senior, one major, and a man in the Indian Medical Department. This, too, was common property. She had 'stayed down three hot weathers' as the saying is, because her brother was in debt and could not afford the expense of her keep at even a cheap hill station. Therefore her face was white as bone, and in the centre of her forehead was a big silvery scar about the size of a shilling, the mark of a Delhi sore, which is the same as a 'Baghdad date'; this comes from drinking bad water. . . . None the less William had enjoyed herself hugely in her four years. Twice she had been nearly drowned while fording a river on horseback, once she had been run away with on a camel, had witnessed a midnight attack of thieves on her brother's camp; had seen justice administered with long sticks in the open under trees, could speak Urdu and even rough Punjabi with a fluency that was envied by her seniors, had altogether fallen out of the habit of writing to her aunts in England or cutting the pages of the English magazines; had been through a very bad cholera year seeing sights unfit to be told, and had wound up her experience by six weeks of typhoid fever, during which her head had been shaved, and hoped to keep her twenty-third birthday that September. It is conceivable that her aunts would not have approved of a girl who never set foot on the ground if a horse were within hail, who rode to dances with a shawl thrown over her skirt, who wore her hair cropped and curling all over her head, who answered indifferently to the name of William or Bill . . . who could act in amateur theatricals, play on the banjo, rule eight servants and two horses, their accounts and their diseases, and look men slowly and deliberately between the eyes—yea, after they had proposed to her and been rejected." She and Scott are in love with each other, and the "motif" of the story lies in the manner in which they both subordinate their feelings to their first duty of rescuing the starving.

Finally the famine is mastered, just as Scott breaks down from overwork. His recovery, however, is rapid, and he and "William" come to an understanding and return to Lahore engaged. There is little plot in the story; all the interest centres on the two finely-drawn characters, "William" and Scott. "William" is a type of the fearless English girl, single-minded and single-purposed, she sees her duty clearly before her, and does it without a thought of self, whether it is to remain by her brother in the sweltering heat of the plains at the expense of her health and appearance, or to sacrifice herself amongst the repellent surroundings of a famine. No less unquestioning are Scott's ideas of duty. To his work, to the needs of the starving population, all his personal feelings and inclinations are made subservient, he overworks himself as a matter of course and unquestioningly until he breaks down. How common the type is in India among Civil servants, both covenanted and uncovenanted, is little known to those who only know India from books or from a hurried cold weather tour; but it is a common type for all that, and the account of Scott working and toiling day and night, organising, arranging, and superintending, and at the same time carrying out with his own hands any menial work that seems necessary to him, might be written a thousand times with real names and real details, and leave many records of such work unrecognised.

Perhaps the cleverest part of the whole story are the few lines descriptive of Lahore in the midst of the hot weather, when the heat has affected all the unfortunate white men left in the plains in mind, body, and nerves, and the bright contrast of the train bringing in the holiday-makers from all parts of the Punjab for the Christmas week. At Christmas Lahore holds high festival, and few as are the lines devoted to the description no one who has spent a Christmas there can fail to see as he reads the lines the old capital of the Punjab before him again, with all its varied sights and smells as the train rolls in through the morning mist.

Lifelike also are the portraits of Jim Hawkins the civilian (whose highest reward and compliment to a good man are "to work him hard"), and his wife, interested, heart and soul, in her husband's work. The same single-minded devotion to duty and the work in hand appears in the "Bridge Builders" also. Into this story Kipling has introduced a meeting of the Hindu gods, who in the shape of animals meet on the half-submerged island on which the river has cast Findlayson the Engineer, and his right-hand native subordinate, Peroo. The fancy for making his animals talk seems to grow upon the writer. Two of the tales in the book are entirely devoted to conversations between horses. He even carries his fancy further, and in one tale gives us an account of conversations between the various portions of a newly-built ship, which have got to understand the reliance they must place on each other before they can work in unison. A similar conceit is given us in the tale called "·007," when long discussions take place between various locomotive engines. "·007" is the name of a newly-built engine which is not admitted to the brotherhood or guild of the engines until it has shown its mettle by taking a relief train to the scene of an accident.

* "The Day's Work." By Rudyard Kipling. (London: Macmillan and Co. 1898.)

### The Need of Nursing Reform in Workhouse Infirmaries.

AN able paper on this subject was read on the 20th inst. by Miss E. E. Julian, matron of the Croydon Union Infirmary, at the general meeting of the London and District Poor Law Officers' Association, held at Hackney. After quoting authorities to prove that unless something were done to relieve the acute difficulty of obtaining nurses in the workhouses the Local Government Board order of 1897 "must become a dead letter," and declaring that there were plenty of suitable nurses to fill the well-paid, responsible posts under the Poor Law, Miss Julian proceeded to discuss the reasons why they kept aloof. The chief objections to fully qualified nurses accepting these positions were, in her opinion, the loss of professional status, the lack of adequate assistance, and the monotonous and uncongenial surroundings. In the opinion of the public the Poor Law superintendent nurse is one who lacks sufficient skill and refinement to become a hospital matron. The position, therefore, in a country district leads neither to professional advancement, nor does it confer social privileges. She has not adequate subordinate assistance, because assistant nurses are disappearing; the younger women know that a certificate is necessary for promotion, and the older women, disliking innovations, seek other work, becoming district, private, or monthly nurses. Miss Julian then compared the difference between the life and surroundings of the superintendent nurse and those of the provincial hospital matron. The one is ignored, the other welcomed by the local gentry; the one is left to her own devices, the other has more invitations than she can accept, more books than she can read, plenty of flowers and companionship. Under such conditions it is not to be wondered at if, after a few months' trial of a superintendent nurse's work, the well-qualified women throw up their posts and warn their friends against accepting similar ones. To meet these detrimental conditions two suggestions have been put forth—the one, by Miss Louisa Twining, that Poor Law nursing should be a State department like the Army and Navy nursing services; the other, by Miss Gibson, that the larger infirmaries should be used as training schools for the smaller infirmaries. Miss Julian then brought forward Miss Wilkie's suggestions, which she made at the Poor Law Conference held in Yorkshire last November, namely, that a uniform standard of training should be adopted, and directed by a nursing department of the Local Government Board; that the period of training should be four years, a portion of the time being spent in the smaller infirmaries; that no salary should be paid for the first two years; that scholarships should be offered in order that really suitable women without means should not be debarred from training. In Miss Julian's opinion, the true test of a certificate is its commercial value, or, in other words, the "position its holder can command in the nursing world," and therefore she commended the idea of the certificate being granted by the Local Government Board. After pointing out that the nurses trained by the best Poor Law institutions have taken their places in rank with those trained in the hospitals, and declaring that the Poor Law authorities must be in the van and not the rear of progress, Miss Julian met two main objections urged against the superior training of the Poor Law nurse. The first, "that such nurses would not remain in the service of the Guardians," by asking if the Poor Law officials kept their nurses now? And why not? Because, she said, outside the training schools, which are virtually hospitals, the "conditions of service are notoriously unsatisfactory." And the second, "that few women would give their service for two years or would pay training fees," by pointing out that in all trades the worker gives either time or money for instruction. If the teaching were thorough, a better and better class of women would come forward, be the terms of engagement what they might. Miss Julian does not advocate the institution of more training schools, but rather the perfecting of those already existing by the institution of courses of instruction in cookery, domestic economy, and practical housewifery. She would, however, like to see preliminary schools on the pattern of Tredegar House inaugurated, in order to eliminate the unfit. For this purpose the smaller workhouses could be utilised, and much valuable time saved in the recognised schools. Miss Julian concluded her paper by enumerating the reasons why small infirmaries could not be regarded as satisfactory training centres. These are: The smallness of the staff, the laxer discipline, lack of properly-equipped class rooms, long hours of service, and the removal of the more serious cases to the larger hospitals because of the non-residence of medical officers.

### Minor Appointments.

ELLEN BADGER MEMORIAL COTTAGE HOSPITAL, SHIPSTON-ON-STOUR.—Miss Ellen E. Wilson was appointed Matron of above hospital on December 6th. She was trained at St. Marylebone Infirmary, afterwards taking up private and Queen's nursing. She has lately held the posts of superintendent nurse at Romford Infirmary and of night superintendent at Camberwell Union Infirmary, East Dulwich, which latter she resigned to accept the above position in her native town.

GENERAL INFIRMARY, LEEDS.—Miss Edith Addis was appointed Assistant Superintendent of Nurses here on January 20th, 1899. She was trained at St. Thomas's Hospital, London, and at Bolton Union Infirmary. Miss Addis is at present night superintendent of the Radcliffe Infirmary, Oxford, where she has previously had charge of male and female blocks, housekeeping, and the dispensary.

CLAYTON HOSPITAL, WAKEFIELD.—Miss Fannie Smithies, who was trained at Bolton Infirmary, was appointed Night Superintendent of this infirmary on the 23rd inst. Miss Smithies has been for three years engaged in private nursing, for four years sister of the accident and surgical ward at the Cardiff Infirmary, and nurse matron of the Bridgend Cottage Hospital for eight months.

ROYAL HOSPITAL FOR SICK CHILDREN, EDINBURGH.—Miss Beatrice M. Lawson has been appointed Assistant Matron of this institution. She was trained at the Dumfries and Galloway Infirmary, N.B., where she has also been head nurse, night superintendent, and, for the last three years, assistant matron.

LANCASTER CORPORATION SANATORIUM.—Miss Ella B. Peters was appointed Certificated Nurse of this institution on the 16th inst. She was trained at the Fever Hospital, Middlesborough, and has been employed by the Kemerton Rural Nursing Association, and at the Royal Military Infectious Diseases Hospital, Aldershot.

ST. OLAVE'S UNION INFIRMARY, ROTHERHITHE.—On the 18th inst. Miss Mary Froome was appointed Charge Nurse here. She was trained at St. Saviour's Infirmary, East Dulwich; and has been head nurse and assistant matron of the British Home for Incurables, Streatham, from 1895—1899.

ISOLATION HOSPITAL, UPNEY, BARKING TOWN, ESSEX.—On the 17th inst. Miss Phillips was appointed Head Nurse here. She was trained at Sir Patrick Dun's Hospital, Dublin, and has also held a post in the Wolverhampton Borough Hospital.

EASTERN HOSPITAL, THE GROVE, HOMERTON, N.E.—Miss Emily Lowe has been appointed the Superintendent of Night Nurses here. She was trained and afterwards staff nurse at the London Hospital.

### Everybody's Opinion.

[Correspondence on all subjects is invited, but we cannot in any way be responsible for the opinions expressed by our correspondents. No communication can be entertained if the name and address of the correspondent is not given, as a guarantee of good faith but not necessarily for publication, or unless one side of the paper only is written on.]

### NURSE AND VACCINATION.

"A NURSE" writes: No doubt many of your readers have read (and if they have not read, may I strongly urge them to do so) the most excellent letter of Mrs. Garrett Anderson in the *Times* of January 10th upon the vexed question of vaccination. Thanks to what one cannot but consider a deplorably retrograde piece of legislation, we have put our selves back to the dark ages before Jenner's great discovery, and now the only thing to be done by those who look upon vaccination as a great and vital good is to try and prevent the disastrous results of the new legislation. Mrs. Anderson suggests that such organisations as the Girls' Friendly Society and the Mothers' Union might do much in the good cause by teaching the poorer classes the truth about vaccination, and exposing the fallacies of the anti-vaccinator. It has struck me that it might also be possible for district nurses, and others, to instruct the ignorant in this most important matter. Could not our nurses be thoroughly taught the use of vaccination, the importance of aseptic vaccination, and the proper care of the vaccinated, and then hand on their knowledge to those among whom they work. Such a handing on of the lamp of knowledge would be an inestimable benefit, not only in helping to ward off so terrible a scourge as small pox, but also by teaching folks reason and common sense we might hope once more to emerge into the broad daylight of civilisation from the outer darkness of bigotry and ignorance.

### RECOVERY OF FEES.

"JUSTICE" writes: Will you kindly allow me to bring before you my 21 years' experience as a ladies' nurse on the subject as to "recovery of fees," when a lady is confined prematurely, and her nurse not free to go to her. Many such instances I have known, and one happened to myself quite recently. The husband wrote to the accoucheur asking what he was to pay me. The reply given was that when a nurse engaged herself to a lady, of course she refused every other applicant for that date, and therefore was entitled to her full fee (unless she should still be able to get another case for that month), and that that did not fully recompense her, for she lost her board during the time. I believe the law is the same in a case of miscarriage, though a nurse of nice mind chooses only to ask "half fee," hoping that she might hear of another case. A nurse only wants justice, which your reply to 155 in "Notes and Queries" does not seem to recognise. Only once have I been asked to relinquish my fee if the engagement was broken, as above. Surely a letter written and signed by a lady ought to stand good as our claim, and if anyone is to be the loser, it ought to be those through whom the contract is broken.

***We are certainly most anxious that every nurse should obtain "justice," and when the balance of conflicting claims as to what is just both to nurse and patient is clearly defined, it will be easier to answer queries upon the subject of "recovery of fee." A written agreement, whether it be a letter signed by the patient, or any other document, is needed to enable anyone to understand what arrangements really were made, as either party or both may be under a misunderstanding if it be only verbal. It is true that in the event of breaking a contract the person who breaks it is liable for the loss, but in making a contract it is always permissible to insert a clause guarding against circumstances arising over which the contractor has no control. Very few ladies can afford to risk the loss of six or eight guineas at a time when they have endless expenses, and most of them are inclined to consider that to demand full fees when no service was rendered is unfair. This is also the view of many maternity nurses. Unless there be a spirit of give and take on both sides very unpleasant friction may result, which will probably end in some other nurse being engaged "next time," and the nurse who caused it losing not only one case but the connection that springs up round every satisfied patient. The true basis on which to estimate the fee to be asked is the loss sustained. If the nurse does her best to obtain another case, and fails to do so, there can be no doubt that she ought to have her full fee. But if she is able to fill up her time she should have no claim except for what will cover her loss, *i.e.*, the difference between the first fee and the second.—ED. *T. H.*

### AFFLUENT BREAD-WINNERS.

"A WORKER" writes: May I ask for a small space in the correspondence page of the "Nursing Mirror" to air a grievance and to put forth a protest? In a daily newspaper not long ago I saw a similar "protest" with reference to lady journalists, and the "protestor" condemned the custom of ladies of position and wealth writing society news for the papers, arguing that there were many clever and well-trained lady journalists who depend on the work for a living, and that to take their duties from them meant also taking their daily bread. Now if this applies to any profession at all amongst women it certainly does to that of nursing. How many ladies have made to me some such remark as the following: "A dear friend of mine is a nurse. She is at the So-and-so Hospital, and she loves nursing. She is not obliged to do it, of course—she has private means—but she simply loves it." I should like to know if it ever occurs to these ladies, who no doubt wish to do good, that they are taking the living away from their poorer sisters, who may be equally fond of the work, but who must of necessity do something to live; who may be well educated, and qualified in every way to fill the better posts which those women of independent means take from them, or in any case considerably reduce their chances of success. I should like to suggest that those with independent incomes, who take their places as competitors in the struggle, share their incomes with their poorer sisters, either by paying the money they do not need themselves into the Royal National Pension Fund for Nurses to swell the bonuses, or by laying it out in some other way for the benefit of those less fortunate, who have no prospect before them but one of ceaseless toil up to old age. The profession is overdone now, and it is very hard that those who are obliged to earn their living should have the battle of life made harder by the competition necessarily brought about by so many entering the profession who need not do so. The scheme of Miss B. C. Davis, in her letter in the "Mirror" for December 31st, would be an excellent one to which to devote the money, and if so given it would be a very small compensation for the very real injury constantly being done to the moneyless members of the nursing world.

### Association for Trained Charwomen.

NURSES who live in flats or chambers in London, and depend for their domestic needs upon the services of charwomen, will be glad to hear of an association which was started last summer under the auspices of the Women's Industrial Council, Buckingham Street, Strand. The "Association for Trained Charwomen" was established with the aim of finding employment for women willing to undertake daily household work. The women are thoroughly taught all the details of household cleaning and domestic work by a certificated teacher of domestic economy, the association undertaking to procure well-paid work for them when trained. Application for charwomen, caretakers, or day-servants, who thoroughly understand the work they profess to do, should be made to Miss Shaw, Hon. Secretary A.T.C. Westfield College, Hampstead.

### Presentations.

THE staff at the South Shields Union Infirmary have presented Nurse Ingram with a very handsome travelling trunk on the occasion of her leaving to take up her new appointment as charge nurse at the Dean's Hospital, South Shields.

MISS FINLAY, on resigning the post of lady superintendent of the Newark Hospital, was presented with a handsome afternoon tea service by the nursing staff and servants.

### A Health Resort in Co. Clare.

KILKEE, a charming seaside spot in co. Clare, is rearing its head proudly in competition with foreign watering-places. Until lately it was somewhat difficult of access, but now a very convenient station in the place itself makes it far more convenient. It is not far from Ennis, and is in direct communication with Kilrush and Miletown Malbay. Fishing, shooting, and golfing are among the attractions, and no doubt it must be a very agreeable winter residence for those who do not care for the gaieties of a town, and who prefer to save themselves the little inconveniences attendant upon wintering abroad.

The climate is exceptionally mild and soft, and very favourable to cases of chronic and acute bronchitis, asthma, and throat affections, also to the various forms of rheumatism so common in our country. The two hotels, the West End and Moore's, belong to Colonel Oakes, and are well managed and comfortable. A large concert hall is attached to Moore's, which contributes much to the pleasure of visitors, who may be received at either of these hotels for £2 per week.

Excursions are numerous, such as to Loophead along the magnificent cliffs, which rise several hundred feet perpendicularly from the sea. Another is to Ross Bay and the "Natural Bridges of Ross." Bathing is very good; the bay is sheltered and the beach excellent. Altogether it is a taking little place, and will no doubt become very popular when it is a little better known.

### Notes and Queries.

**The contents of the Editor's Letter-box have now reached such unwieldy proportions that it has become necessary to establish a hard and fast rule regarding Answers to Correspondents. In future, all questions requiring replies will continue to be answered in this column without any fee. If an answer is required by letter, a fee of half-a-crown must be enclosed with the note containing the enquiry. We are always pleased to help our numerous correspondents to the fullest extent, and we can trust them to sympathise in the overwhelming amount of writing which makes the new rules a necessity.**

**Every communication must be accompanied by the writer's name and address, otherwise it will receive no attention.**

*Twelve Months' Training.*

(171) I am anxious to get into an established training hospital for a year, where it is possible to gain a certificate at the end of the twelve months. Could you inform me which is the best one to write to? Also the one requiring the smallest premium. I have written about one, but the money is £25.—*Half-Trained.*

See "Nursing Profession: How and Where to Train," price 2s. net, from the Scientific Press, London.

*Materia Medica.*

(172) Could you kindly tell me if there is any simple pocket dictionary of medicine, telling of what they are composed, and what effect they have in various diseases, &c.?—*Probationer.*

"Materia Medica and Therapeutics," by Dr. Mitchell-Bruce (Cassell's), is an admirable book, but it is not a very little one.

*Stewardess.*

(173) Will you kindly inform me what is the best way to get a post as stewardess on board the P. and O. steamers and address of the office? I find it very difficult to get work in London, having now been weeks without earning a penny, for the nursing profession seems overrun, and, as in most cases, the younger nurses are preferred to the older.—*Miserére.*

Write to the secretary of the P. and O., 122, Leadenhall Street. Have you tried to obtain a permanent elderly patient? The work is sometimes very light, even if the pay is small. See our advertisement columns.

*Case Book.*

(174) I would be glad to know if there is a district nurses' case book in print in which a record of names, addresses, diseases, dates, and little memoranda could be kept?—*Sister Margaret.*

"THE HOSPITAL Nurses' Case Book," price 6d. (Scientific Press, London), would suit you.

*An Idle Girl.*

(175) Can anyone tell me of a training home (in the North of England, if possible) for a troublesome girl of 15, who is continually being sent home from service on account of idleness and untidy habits. She has very respectable but poor parents.—*Nurse R.*

Perhaps some of our readers may help. Failing that, see list "Burdett's Hospitals and Charities" (Scientific Press, London, W., price 5s.) under heading "Orphanages, Homes, and Charities."

*Hysteria.*

(176) Can you tell me of a home where an hysterical patient could be placed? The friends of a patient of mine, with whom I have been over four months, are anxious to find a suitable place for her. She could pay £1 a week, but could not afford more. The case is one of an aggravated chronic nature, but she is in good bodily health and there is really no need for a trained nurse. Her friends would feel greatly obliged to you if you could help them. You have helped me more than once out of my own difficulties through your valuable paper, I know I might venture to ask you on behalf of others.—*Nurse F.*

It is of the utmost importance that hysteria should be under good medical care. Hysteria should be cured and not merely sent to a home. You might perhaps get her into the National Hospital for the Paralysed and Epileptic, Queen's Square, Bloomsbury, where she might (if the case be suitable) become a paying patient at £1 1s. a week.

*Paris.*

(177) Would you kindly let me know if there is a nursing home for trained nurses in Paris, and, if so, the address?—*Nurse MacL.*

The Hollond Institute, 1, Tavistock Chambers, Bloomsbury, W.C., has a branch in Paris.

*Private Mental Nursing.*

(178) Will you kindly favour me with addresses of institutions for private mental nursing?—*F. C.*

We cannot furnish you with the addresses of private mental nursing homes.

*Nursing Abroad.*

(179) Kindly inform me where to apply for information (1) regarding nursing in the Colonies, (2) Lady Dufferin's Nurses for India, (3) Lady Roberts' Nurses for India, (4) also the Army Nursing Service abroad?—*E. J.*

The Colonial Nursing Association, Imperial Institute, S.W. 2. Lady Dufferin's Fund is chiefly to supply medical, not nursing, aid in India. 3. Lady Roberts selects her own nurses, and does not receive outside applications. 4. Army Nursing Service, the Adjutant-General of the Forces, War Office, Pall Mall, S.W.

*Gynæcological Training.*

(180) Will you kindly tell me if there are any hospitals where they give six months' training in surgical nursing or in gynæcology to a nurse who holds a certificate in midwifery and has had only six months' general training?—*E. M. B., Stafford.*

You could only obtain so short a training as a paying probationer. See "Burdett's Official Nursing Directory," for terms and particulars of training.

*A Full Report.*

(181) Will you tell me where I can procure Sir William Broadbent's address on "The Conduct of the Heart in the Face of Difficulties," given at the Medical Society on the 9th inst., and from which you quote in THE HOSPITAL of the 14th.—*Alison.*

We do not think that the address has been printed in full, but an outline appeared in the *Lancet* and the *British Medical Journal* at the same time as the article you refer to.

*Study.*

(182) It has been my great desire and ambition ever since I was a little mite to become a hospital nurse, and as now I am only sixteen, I feel it must be some years before I can fulfil this wish. Would you oblige me by mentioning the subjects I ought to be well up in to pass any nursing examination, at the same time mentioning the best books that would help me to prepare myself for them.—*H. L. B.*

The usual subjects of a good elementary education, reading, writing, arithmetic. Cultivate all your talents as widely as possible, for when you begin your special training there is little time for other things, and it is a drawback for a nurse to be without varied interests. All housewifely arts, cooking, needlework, bookkeeping, music, French, &c., are useful.

*Schools.*

(183) Is the Isle of Wight Infirmary and Ryde County Hospital a recognised school for training, and will a certificate taken there be of the same standing as one from a London hospital, for a nurse who wishes to take up private work after training? and is the Bristol Infirmary a good recognised school?—*L. M.*

There is no training school for nurses at the Royal Isle of Wight Infirmary, Ryde. (2) The Bristol Royal Infirmary is a recognised training school, of 270 beds.

*Hospital Training.*

(184) Will you kindly tell me if it is possible to call Plaistow training at the Maternity Charity and District Nursing Home hospital training, because I cannot see that it is. Our County Council last year gave us scholarships for hospital training for one year, and then we gave four months' maternity at Glasgow as well, and so turned out a very good nurse. Now, because other County Councils do, they want to send them to Plaistow and call it hospital training. Some of my committee favour this, but as I have to find the nurses and follow the rules, which I enclose, how can we call it hospital training if it is not. That it is excellent district nursing and maternity training I have no doubt. For years I have been trying to raise the standard of nursing, which is really very good, but I shall be sorry if, through ignorance, my committee make a mistake. I am sorry to trouble you, but should like the opinion of an independent person.—*Lady Superintendent.*

The Plaistow training is in no sense of the term hospital training. It teaches maternity nursing for district work, but we do not know that the County Councils are limited to *hospital* training.

*Training.*

(185) I am very anxious to have three years' training in a good hospital. I am 24, but only 5 ft. 2 in. Can you tell me if I am likely to succeed? I am now children's nurse.—*C. E. T.* (Salop).

If suitable in other ways there is no reason why you should not succeed. Apply first to the matron of the Salop Infirmary, Shrewsbury.

*Return to Hospital Work.*

(186) A nurse, trained for three years in a well-known training school for nurses and certificated, wishes to return to hospital work again, but having been engaged in private nursing for the last three years, does not know how to do so. Will you kindly inform her if it is possible to get a post in a hospital, also what hospitals, without commencing as probationer again?

See advertisements, of which there are plenty, and apply in the usual way.

### Travel Notes.

By Our Travelling Correspondent.

### VIII.—VILLEFRANCHE, &c.

Where all is beautiful it is very hard to pick out one place as surpassing all others in loveliness, but I have always found my affections returning to Villefranche as the most charming nook along the French Riviera. The accompanying sketch I made by the courtesy of the station-master from the upside of the line. Imagine that scene laid out in colours so brilliant as to be absolutely dazzling—a sky of pure ultra-marine, and a sea of emeralds and sapphires. The bay is very deep, and huge men-of-war can anchor close in under the brown old houses as they climb the rocks above and cling as if by magic to insecure ledges that appear hardly strong enough to support a wheelbarrow. Down in the little harbour itself all is picturesque and primitive; a flight of steps leading from the lower Corniche to the water's edge, narrow and steep, comprises the chief commercial street of Villefranche. Here buying and selling, love-making and quarrelling, are perpetually occupying the leisure of the handsome black-eyed natives. Piles of fruit and vegetables and huge boughs laden with oranges light up all the dim entries, and overflow in rich and abundantly coloured profusion on to the rough steps above and below; in the midst a mule paces daintily up and up, making nothing of the treacherous stair, that is a trial to a high-heeled society dame. On the mule sits secure and at ease his mistress, in the curious flat hat of light straw peculiar to the country. It resembles a Chinaman's, and has no crown at all. Unfortunately, this hat, the last remnant of costume in the Riviera, is fast disappearing. Villefranche is not a place to stay in, except for the robust, enterprising, and daring artist, but is so very easily reached from Nice by rail, carriage, or foot that it seems only like a suburb of that gay and fashionable town. The approach by the Mont Boron road is very impressive; there are diligences that make the journey about every hour with very good horses. I was there last year and encountered suddenly a truly fiendish mistral and a veritable dust storm. The gallant chasseurs were drilling on their parade ground, and I was trying to sketch them when I was really engulphed by sand. The noble warriors rushed to my assistance and covered my sketching things and camera with spare coats.

### Aspremonte, Falicon, and Grotto of St. André.

All these three excursions are easy and can be done by carriage. The two latter can be managed together. You pass by the monastery of St. Pons, said to have been commenced in 775. It has been twice destroyed and rebuilt. The prettiest part of the road is that between the Grotto and Falicon. To make this excursion pleasurably and without over fatigue, it is well to start early and rest well in the middle of the day. Aspremonte is much nearer, and is picturesquely situated, though not so remarkably as Eze or St. Agnèse, Peille, or Roccabruna; but it is so easy of access, and is suitable for early convalescence.

### Avoid Fatigue for Invalids.

In your ardour to amuse your charge, do not overdo him. Still, in a thoroughly comfortable carriage such as the Nice hotels provide, the fatigue is not much, and by having a light stretcher made by an ordinary carpenter of a few laths, put across the seats, with a small mattress comfortably placed on it, your invalid can lie down or not just as is desirable, in the same way as if he was at home. The refreshment provided in these little mountain places is by no means to be relied on, and it is better to take a luncheon or tea basket with you, with everything necessary therein, so that you can remain out the entire day if you think it well. Often a capricious invalid, who drives you to the verge of distraction with his daintiness at home will make a good pic-nic meal—amused and carried out of himself by the novel surroundings. While the horses are being rested, tell him to go to sleep; well wrapped up there is no danger in sleeping in the open air—quite the contrary, if the slumberer is well wrapped up and the sun is still high in the heavens. I am, perhaps, an enthusiast for the fresh air cure, because I have seen such marvellous results from simply taking the remedy which God has put into our hands; and if we are not going to use it, why take this long journey only to shut one's self up in an hotel. This is a very good bicycle road, and if any of your party are votaries of the wheel, about 20 miles takes you there and back.

Villefranche.

### Eze (Eza).

Eze is quite one of the most striking points on the Riviera; it lies between Nice and Monte Carlo and above Beaulieu. As I before remarked it is impossible to drive all the way; there are four or five ways of reaching this jealously-guarded fortress town, once a stronghold of the Saracens, but by all, much or little walking is required. It is so wonderfully placed in its grand isolation, that at first one wonders if it is possible to reach it, perched like an eagle's nest far above us or below, according to the route by which we have come. It is 1,300 feet above sea level, but several hundreds below the Corniche. The easiest way to go from Nice is by driving along the Corniche and getting out at a road which goes straight down into Eze; the walk would make two miles each way, and the return would naturally be the more fatiguing. If the sturdy mean to attempt the excursion unhampered by invalids, almost the prettiest way is by train to Eze station, and then slowly to mount the heights; it will take you about an hour and a quarter, and is rather a pull, but the beauties you encounter make you forget all fatigue. A third way, by which all climbing is avoided, is by taking the train to Monte Carlo, then the mountain railway to La Turbie, a most interesting spot of which I will speak later on, and then to walk along the Corniche until you come upon a mule track which will lead you straight into Eze. Having seen the little town continue the mule track down to Eze Station, and take the train home. It is indescribably lovely all round; the grandeur of the approach to Cap Roux from the lower Corniche cannot be surpassed, the huge rock descends sheer into the sea, and the railway passes through it by a tunnel.

### Petite Afrique.

This part of the coast is called Petite Afrique, because it is so extremely sheltered that the rich vegetation, semi-tropical in its character, here flourishes more luxuriantly than on any other part of the Riviera. As one descends to Eze Station on the edge of Petite Afrique and glances back up to Eze, one marvels how the builders ever accomplished their task; the houses, forts, and walls appear to cling like ivy to the rock; and at so giddy a height how awesome must have been the builder's duties. It must in olden times have been well nigh impregnable, and how Barbarossa managed to overthrow it in the manner he did is a puzzle. All now is desolate and decayed, and I have often wondered what the lives of those shut up in these fortress-like places, ruined and poverty-stricken, can be like. What is their occupation? How do they live? Does joy and sorrow, peace and strife come there, and do births, deaths, and marriages occur there as elsewhere? One must suppose so, but it hardly seems probable.

### TRAVEL NOTES AND QUERIES.

Rules in regard to Correspondence for this Section.—All questioners must use a pseudonym for publication, but the communication must also bear the writer's own name and address as well, which will be regarded as confidential. All such communications to be addressed "Travel Editor, 'Nursing Mirror,' 28, Southampton Street, Strand." No charge will be made for inserting and answering questions in the inquiry column, and all will be answered in rotation as space permits. If an answer by letter is required, a stamped and addressed envelope must be enclosed, together with 2s. 6d., which fee will be devoted to the objects of the "Hospital Convalescent Fund." Any inquiries reaching the office after Monday cannot be answered in "The Mirror" of the current week.

Through Brittany (Vespers).—It would not be advisable to start anywhere in Brittany earlier than May, because though the climate is milder than that of England, the hotels (off the beaten track) are primitive, and you would feel the lack of comforts. Write me again later on.

Bruges (Sheraton).—I hardly think this town will meet your views. It is too damp on account of the canals to be suitable for an invalid. You would find Brussels drier and more cheerful, or perhaps Dinan, in Brittany, which is milder than Brussels, would do. Dinan is cheap. I think you might live *en pension* there comfortably for six francs a day.

Riviera (Tommy Atkins).—You do not tell me what you can afford, but I gather that it is necessary to be economical. At Nice try for rooms at the Hotel Suisse, but you must apply very early. Cannes is quite as expensive as Nice. Try the Hotel Royal, pension from nine to twelve francs. Mentone is, perhaps, a trifle cheaper, and is a delightful spot. The Hotel Sta Maria is very good, with terms from eight to twelve francs. San Remo is not so fashionable as Nice and Cannes, but has great charms of its own. Try for rooms on the third floor at Hotel Nationale, eight to twelve francs.

Naples (Anglo-Indian).—Quickest route via Dover, Bale, Milan, and Rome. First-class, £12 2s. 6d.; second, £8 10s. Hotels rather expensive, and not advisable to risk second-class houses.

Coblentz (Hap hazard).—I think it lies a little low for your purpose. Why not go to Heidelberg, and stay up on the wooded hill above the Castle. The hotel is called the Schloss.

Dolomites (Busby).—I do not think it a country suited to your needs. Though most lovely and healthy, the journey is fatiguing, and as yet the arrangements are somewhat primitive, and not what are required for those who are delicate. Why not try the Pyrenees? San Jean de Luz is excellent for rheumatism, and is more suited to those suffering from chest trouble.

Florence (Aurora).—Yes, it is cold, but dry and clear, with plenty of sun. You can remain there comfortably till May, when it would be very agreeable to move up to the Italian lakes for the months of May and June, then on to the high Alps for July and August and part of September, coming down to Florence or Rome by October.

Florence (One Summer's Day).—Read the answer to "Aurora." Yes, you can find masters for everything, and at reasonable rates.

### For Reading to the Sick.

### REVERENCE.

### Verses.

A holy awe
For holy things; not those that men call holy,
But such as are revealèd to the eyes
Of a true woman's soul bent down and lowly
Before the face of daily mysteries. —*Lowell.*

Unparallel'd in any age or land,
Fair fame, bright honour, virtue firm, rare grace,
The chastest beauty in celestial fame—
These be the roots whence birth so noble came.
Such ever in my mind her form I trace,
A happy burden and a holy thing,
To which on rev'rent knee with holy prayer I cling.
—*Petrarch.*

The vision which we have
Revere we so,
That yet we crave
To foot those fields of ne'er profaned snow.
—*Coventry Patmore.*

Deliver not the tasks of might
To weakness, neither hide the ray
From those, not blind, who wait for day,
Tho' sitting girt with doubtful light.

Make knowledge circle with the winds;
But let her herald, Reverence, fly
Before her to whatever sky
Bear seeds of men and growth of minds!
—*Tennyson.*

### Reading.

The deeper movements of the soul shrink back from our imputations, refuse to be made the tools of our prudence, and insist on coming unobserved, or coming never; and he that reckons on them sends them into banishment, and only shows that they are and must be strangers to his barren heart.

Of nothing may we be more sure than this—that if we cannot sanctify our present lot we could sanctify no other. Our Heaven and our Almighty Father are there or nowhere. The obstructions of that lot are given for us to heave away by the concurrent touch of a holy spirit and labour of strenuous will; its mysteries are for our worship; its sorrows for our trust; its perils for our courage; its temptations for our faith.

Whatsoever is most deep within us is the reflection of Himself. All our better love and higher aspirations are the answering movements of our nature in harmonious obedience to His Spirit.—*Martineau.*

So wonderful is love and so immeasurably important is its influence on mental life! We glorify love, we reverence in it the most powerful factor in human civilisation.—*Haeckel.*

We are to dignify to each other the daily needs and offices of man's life, and embellish it by courage, wisdom, and unity.—*Emerson.*

In reverence is the chief joy and power of life.—*Ruskin.*

Character is far more an inspiration than a manufacture. Toil of discipline and patience, of culture, may accomplish wonders in shaping a soul; but the outlook of a reverent love to a nobler nature will draw down into the inner springs of the being the forces of that better life, and they will move from within in deeper breathings and fuller pulsings of the spirit. A deep true love will lift a soul out of the shallows of selfishness and the mud of fleshliness, when all other powers combined have failed to extricate it from the slough.—*Heber Newton.*

## Figures and Tables

**Figure f1:**